# Efficacy of shear wave elasticity for evaluating myocardial hypertrophy in hypertensive rats

**DOI:** 10.1038/s41598-021-02271-6

**Published:** 2021-11-24

**Authors:** Yoichi Takaya, Kazufumi Nakamura, Rie Nakayama, Hiroaki Ohtsuka, Naofumi Amioka, Megumi Kondo, Kaoru Akazawa, Yuko Ohno, Keishi Ichikawa, Yukihiro Saito, Satoshi Akagi, Masashi Yoshida, Toru Miyoshi, Hiroshi Ito

**Affiliations:** 1 Department of Cardiovascular Medicine, Okayama University Graduate School of Medicine, Dentistry and Pharmaceutical Sciences, 2-5-1 Shikata-cho, Kita-ku, Okayama, 700-8558 Japan; 2 Kawasaki University of Medical Welfare, Okayama, Japan

**Keywords:** Cardiology, Medical research

## Abstract

Shear wave (SW) imaging is a novel ultrasound-based technique for assessing tissue characteristics. SW elasticity may be useful to assess the severity of hypertensive left ventricular (LV) hypertrophy. This study aimed to evaluate the efficacy of SW elasticity for assessing the degree of myocardial hypertrophy using hypertensive rats. Rats were divided into hypertension group and control group. SW elasticity was measured on the excised heart. Myocardial hypertrophy was assessed histologically. LV weight was greater in hypertension group. An increase in interventricular septum and LV free wall thicknesses was observed in hypertension group. SW elasticity was significantly higher in hypertension group than in control group (14.6 ± 4.3 kPa vs. 6.5 ± 1.1 kPa, P < 0.01). The cross-sectional area of cardiomyocytes was larger in hypertension group than in control group (397 ± 50 μm^2^ vs. 243 ± 14 μm^2^, P < 0.01), and SW elasticity was positively correlated with the cross-sectional area of cardiomyocytes (R = 0.96, P < 0.01). This study showed that SW elasticity was higher in hypertensive rats and was closely correlated with the degree of myocardial hypertrophy, suggesting the efficacy of SW elasticity for estimating the severity of hypertensive LV hypertrophy.

## Introduction

Shear wave (SW) imaging is a novel ultrasound technique for assessing characteristics of tissues based on SW propagation velocity^1–3^. SW is generated as propagating wave by pushing pulse of ultrasound beam, which deforms a part of the tissue. SW velocity within the tissue is detected by tracking pulse. SW imaging provides information regarding tissue elasticity, which is calculated by SW velocity.

SW imaging has been used for evaluating several organs, including the liver, breast, and thyroid^4–9^. SW elasticity has been reported to be correlated with tissue stiffness in liver diseases^4,5^. In the field of cardiac diseases, some studies reported that SW elasticity could be used to measure myocardial stiffness in animal models^10–12^. In a clinical setting, one study reported that SW elasticity was higher in patients with hypertrophic cardiomyopathy^13^. However, hypertrophic cardiomyopathy has remarkable left ventricular (LV) hypertrophy. It remains unclear whether SW elasticity can assess the severity of hypertensive LV hypertrophy.

Hypertensive LV hypertrophy is a common cause of heart failure, leading to adverse clinical outcomes^14^. The assessment of LV hypertrophy is beneficial for diagnosing disease severity and considering therapeutic strategies. We hypothesized that SW elasticity reflects the degree of myocardial hypertrophy. This study aimed to evaluate the efficacy of SW elasticity for assessing myocardial hypertrophy of left ventricle using hypertensive model rats.

## Methods

### Animal experiments

Dahl salt-sensitive rats develop hypertension due to a high-salt diet. Adult male Dahl salt-sensitive rats (Japan SLC, Shizuoka, Japan) were purchased and housed under conditions of constant temperature (22 ℃) and humidity (60%), exposed to a 12-h light/dark cycle, and offered tap water to drink. Rats were divided into two groups: hypertension group (n = 6), which were fed a high-salt (8% NaCl) diet for 9 weeks to develop LV hypertrophy, and control group (n = 6), which were fed a normal diet. Systolic blood pressure was measured at 9 weeks by tail-cuff plethysmography (MK-2000; Muromachi, Japan), and the average value of three measurements was calculated. All surgery was performed under inhalation of 2% isoflurane anesthesia, and all efforts were made to minimize suffering. All protocols for animal experiments were approved in accordance with the recommendations of the Okayama University Animal Care and Use Committee, and all methods were performed in accordance with the relevant guidelines and regulations. This study was reported in accordance with the ARRIVE guidelines.

### Echocardiography

Transthoracic echocardiography was performed with Aplio ver. 6.0 with a 10-MHz sector probe (Canon Medical Systems, Otawara, Japan) using gel for ultrasound. The rats were anesthetized from inhalation of 2% isoflurane while lying in a left recumbent position, and were warmed to their body temperature. LV end-diastolic and end-systolic diameters were measured in the short-axis view, and fractional shortening was calculated. Interventricular septum and LV free wall thicknesses were measured (Supplementary Fig. S1).

### Shear wave elasticity

SW imaging was performed by ex vivo experiment. A retrograde perfusion system was used to maintain the rat’s heart in a completely relaxed state^15^. After sacrifice under inhalation of 2% isoflurane anesthesia, the heart was quickly excised and submerged in the Tyrode solution (136 mmol/L NaCl, 5.4 mmol/L KCl, 1.8 mmol/L CaCl_2_, 0.53 mmol/L MgC1_2_, 5.5 mmol/L HEPES, and 1% Glucose, pH 7.4, 37℃) with 20 mmol/L butanedione monoxime, an inhibitor of actin-myosin interaction, and 10 μmol/L blebbistatin, a specific myosin II inhibitor. The ascending aorta was cannulated with an 18-gauge blunted needle connected to a retrograde perfusion system. The heart was perfused with the Tyrode solution with butanedione monoxime and blebbistatin to induce complete relaxation. The heart was set in a water tank of agar phantom.

**Figure 1:**
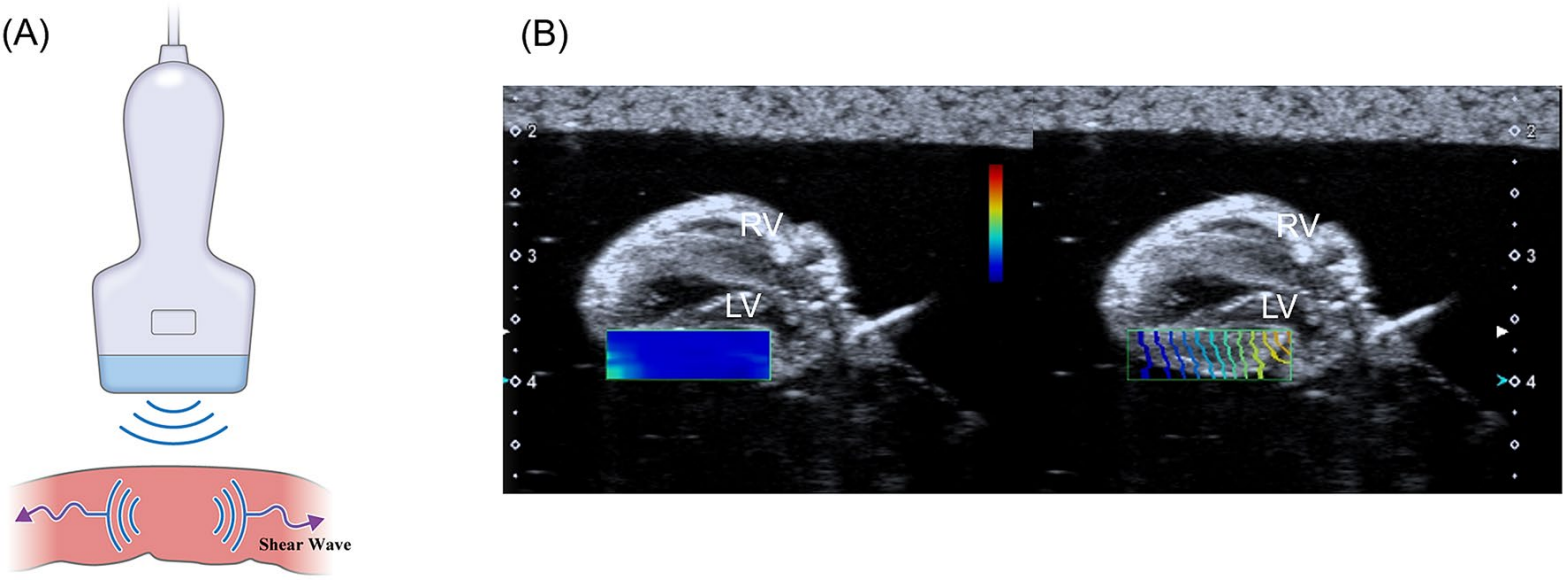
SW imaging. (**A**) SW was generated by pushing pulse of ultrasound beam. (**B**) SW propagation on left ventricular free wall. SW elasticity was automatically measured. *LV* left ventricle, *RV* right ventricle, *SW* shear wave.

SW elasticity was measured with Aplio i900 with an 18-MHz linear probe (Canon Medical Systems). B-mode image was obtained in the long-axis view. A rectangular region of interest was placed on LV free wall. SW was generated by pushing pulse, and SW velocity was obtained based on the tissue Doppler technique (Fig. 1). After confirming a proper SW propagation in “wave front” style display, a circular region of interest of 1-mm in diameter was placed on the image. SW elasticity was measured automatically using the equation: 3*ρc*^*2*^ (*c*: SW velocity, *ρ*: tissue density). Each measurement was repeated five times, and the average value was calculated.

### Histology

The heart was sectioned transversely at the mid-papillary level, and then fixed with 10% formalin, embedded in paraffin, and cut into 5-μm-thick sections. For morphological analysis, sections were stained with hematoxylin–eosin. Samples were immersed in xylene and alcohol, stained with hematoxylin for 7 min, stained with eosin for 4 min, and re-immersed in alcohol and xylene. For detecting fibrosis, sections were stained with picrosirius red using the Picrosirius Red Stain Kit (Polysciences, Inc., Warrington, PA, USA). Samples were immersed in xylene and alcohol, stained with phosphomolybdic acid for 2 min, stained with Picrosirius Red F3BA Stain solution for 1 h, stained with hydrochloride acid for 2 min, and re-immersed in alcohol and xylene. The sections were examined with light microscopy. The cross-sectional area of 50 individual cardiomyocytes on LV free wall in five sections within the heart was measured in the regions containing cellular nucleus using Image J software (version 1.52v, National Institutes of Health, Bethesda, MD, USA) (Supplementary Fig. S2), and the average value was calculated. Interstitial fibrosis on LV free wall was assessed using computer-assisted image analysis^16,17^, and the percentage of fibrosis area was calculated using Image J software (Supplementary Fig. S3).

### Statistical analysis

Data are presented as mean ± standard deviation for continuous variables. Variables were compared by unpaired t-test. Relationships of SW elasticity with myocardial hypertrophy and fibrosis were analyzed by Pearson’s correlation coefficient. Intra- and inter-observer variability was analyzed using Bland–Altman method. The measurements of SW elasticity were evaluated by two blinded observers and by a single observer at two different times. Statistical analysis was performed with JMP version 14.2 (SAS Institute Inc., Cary, NC, USA), and significance was defined as a value of P < 0.05.

## Results

### Baseline characteristics

Baseline characteristics are shown in Table 1. Body weight was significantly lower in hypertension group than in control group. Heart weight and LV weight were greater in hypertension group than in control group. Systolic blood pressure was higher in hypertension group than in control group. An increase in LV end-diastolic and end-systolic diameters with a decrease in fractional shortening was observed in hypertension group. Interventricular septum and LV free wall thicknesses were increased in hypertension group.

**Table 1:** Baseline characteristics.

Variables	Hypertension group (n = 6)	Control group (n = 6)	P
Body weight (g)	272 ± 15	338 ± 8	< 0.01
Heart weight (g)	1.6 ± 0.1	1.4 ± 0.1	< 0.01
LV weight (g)	1.2 ± 0.1	0.9 ± 0.1	< 0.01
Systolic blood pressure (mm Hg)	184 ± 1	142 ± 1	< 0.01
**Echocardiography**			
LV end-diastolic diameter (mm)	8.4 ± 0.4	6.8 ± 0.3	< 0.01
LV end-systolic diameter (mm)	5.7 ± 0.6	3.2 ± 0.7	< 0.01
Fractional shortening (%)	32 ± 7	54 ± 9	< 0.01
Interventricular septum thickness (mm)	1.4 ± 0.1	0.9 ± 0.1	< 0.01
LV free wall thickness (mm)	1.5 ± 0.1	1.1 ± 0.2	< 0.01

Data are presented as mean ± standard deviation.

*LV* left ventricular.

### Shear wave elasticity

**Figure 2:**
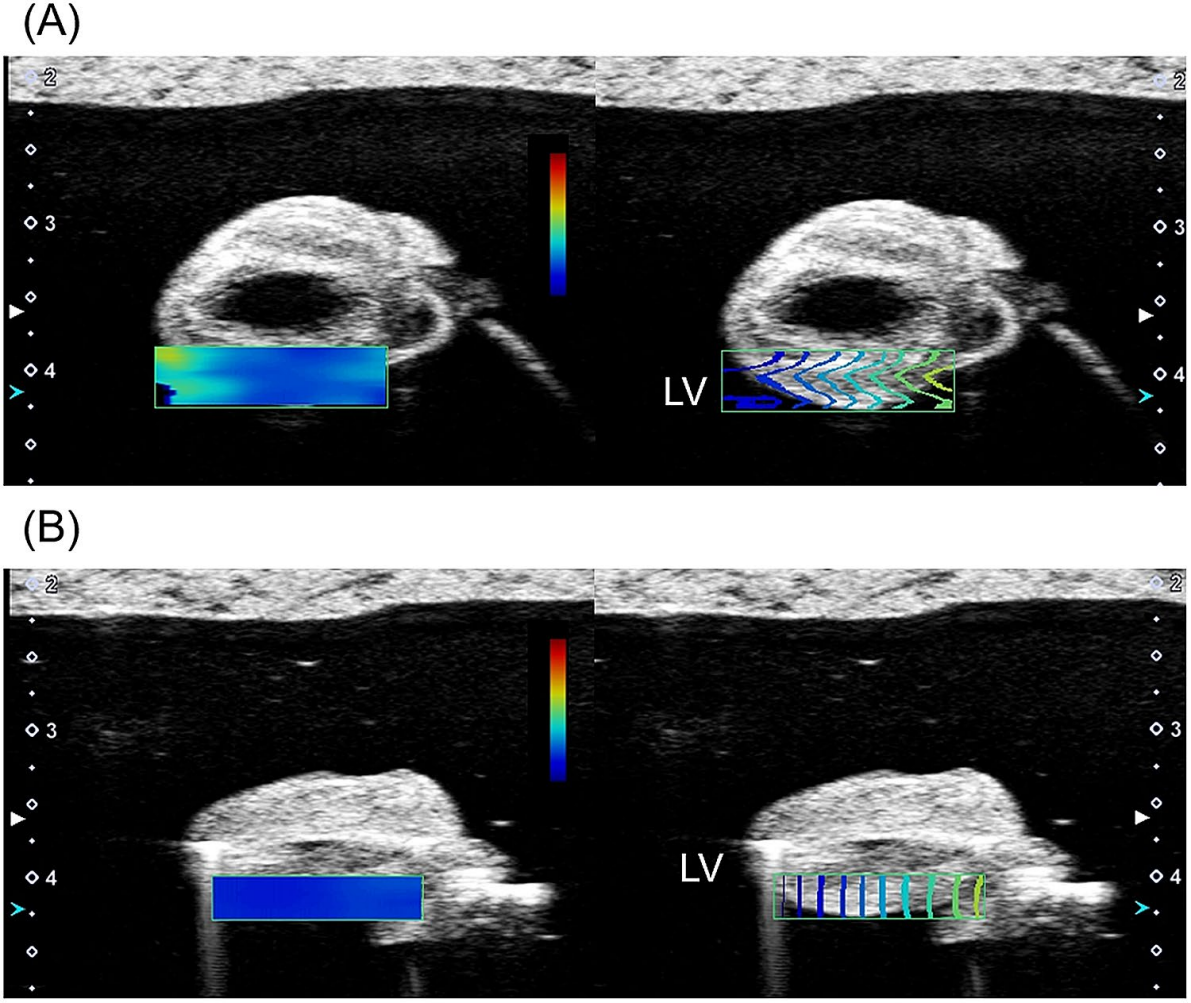
Representative cases of SW imaging. SW imaging in a hypertensive rat (**A**) and a control rat (**B**). *LV* left ventricle, *SW* shear wave.

**Figure 3:**
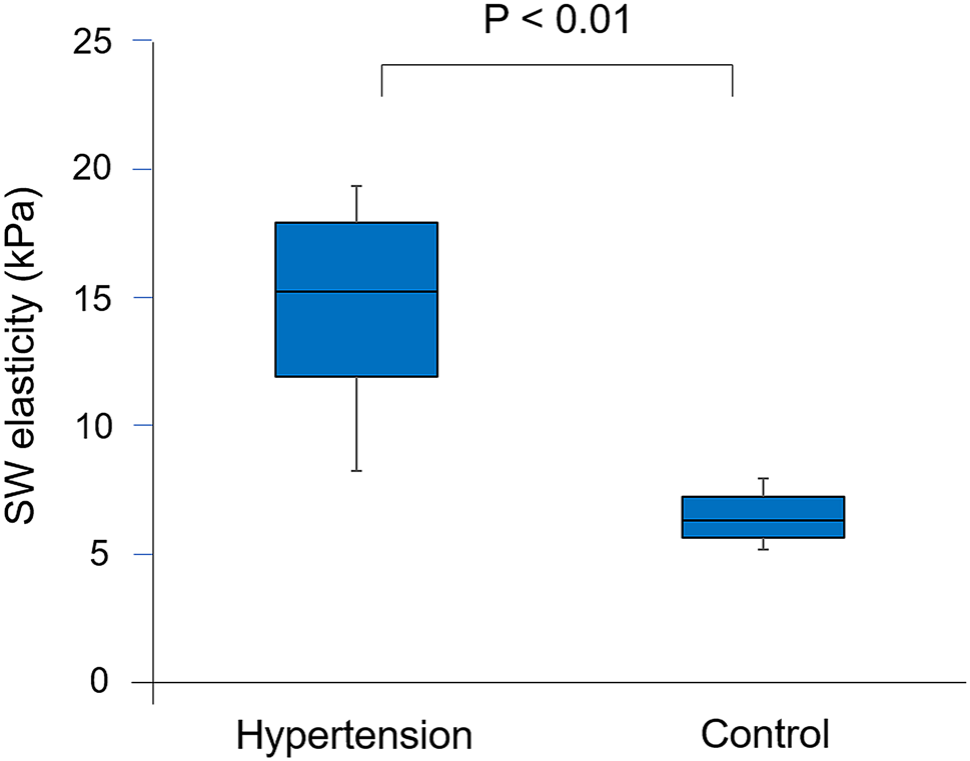
Comparison of SW elasticity. SW elasticity was higher in hypertension group than in control group. *SW* shear wave.

Representative cases in hypertension group and control group are shown in Fig. 2. SW elasticity was 15.5 kPa in a hypertension rat and 6.2 kPa in a control rat. Comparison of SW elasticity of left ventricle between hypertension group and control group is shown in Fig. 3. SW elasticity was significantly higher in hypertension group than in control group (14.6 ± 4.3 kPa vs. 6.5 ± 1.1 kPa, P < 0.01).

### Histological assessment

**Figure 4:**
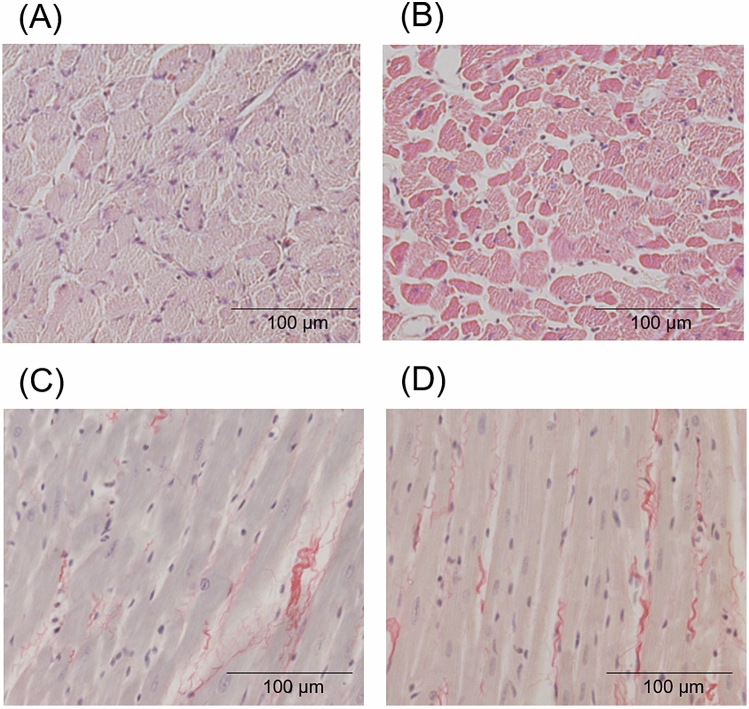
Histological findings. Histological findings stained with hematoxylin–eosin in a hypertensive rat (**A**) and a control rat (**B**). The cross-sectional area of cardiomyocytes was measured. Histological findings stained with picrosirius red in a hypertensive rat (**C**) and a control rat (**D**). Scale bar indicated 100 μm.

Hematoxylin–eosin staining of left ventricle in hypertension group and control group is shown in Fig. 4A,B. The cross-sectional area of cardiomyocytes was significantly larger in hypertension group than in control group (397 ± 50 μm^2^ vs. 243 ± 14 μm^2^, P < 0.01). Picrosirius red staining of left ventricle in hypertension group and control group is shown in Fig. 4C,D. The amount of myocardial fibrosis was small, and the percentage of fibrosis was not different between hypertension group and control group (2.1 ± 1.1% vs. 1.7 ± 0.2%, P = 0.40).

### Relationship of shear wave elasticity with myocardial hypertrophy

**Figure 5:**
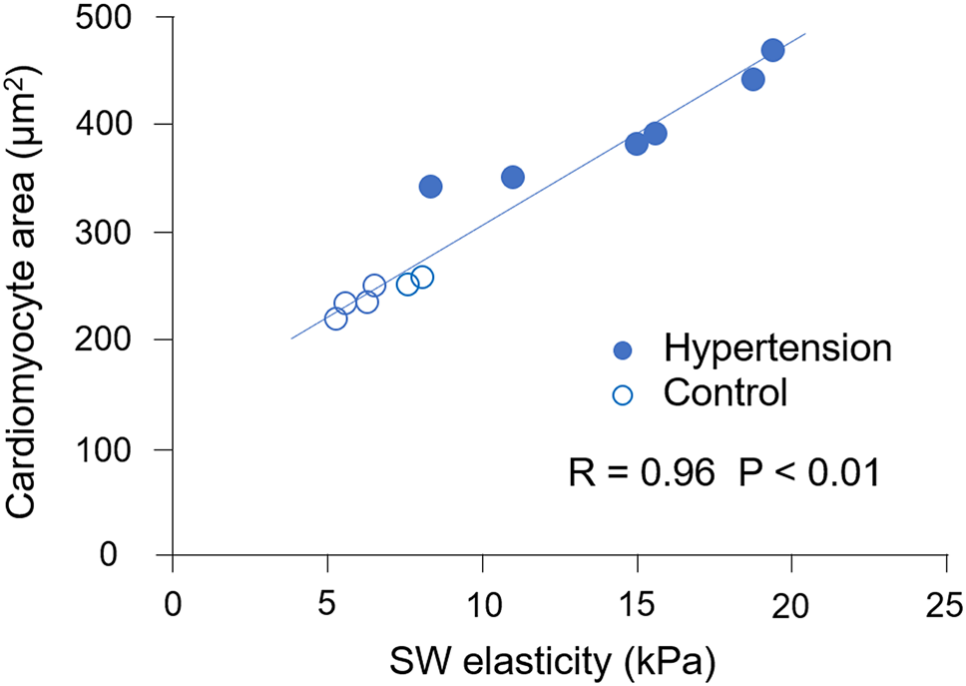
Relationship of SW elasticity with myocardial hypertrophy. SW elasticity was correlated with the cross-sectional area of cardiomyocytes. *SW* shear wave.

SW elasticity was positively correlated with the cross-sectional area of cardiomyocytes (R = 0.96, P < 0.01) (Fig. 5). There was no correlation between SW elasticity and the percentage of myocardial fibrosis.

### Reproducibility

For the intra-observer variability for the measurements of SW elasticity, Bland–Altman method showed the mean difference of − 0.05 kPa (95% confidence intervals of limits of agreement, − 0.11 to 0.02 kPa). For the inter-observer variability, Bland–Altman method showed the mean difference of 0.02 kPa (95% confidence intervals of limits of agreement, − 0.09 to 0.13 kPa).

## Discussion

The major findings of the present study were: (1) SW elasticity was higher in hypertensive rats; and (2) SW elasticity was positively correlated with myocardial hypertrophy. To the best of our knowledge, this is the first study to show the efficacy of SW elasticity for assessing the degree of myocardial hypertrophy of left ventricle.

The severity of LV hypertrophy is associated with adverse outcomes in the clinical setting^14^. LV hypertrophy is a common cause of heart failure with preserved LV ejection fraction, resulting in hospitalization. LV hypertrophy involves changes in myocardial tissue. Assessing the degree of myocardial hypertrophy can be useful for identifying the disease severity in the hypertensive population.

SW imaging is a novel technique for characterizing tissue structures by ultrasound beam. SW elasticity clarifies the contrast of anatomical structures consisting of tissues with different shear coefficients, thereby enabling tissue identification^1,18^. Although SW elasticity has been recognized as a useful tool for characterization of liver diseases^4,5,19–21^, the efficacy is limited in cardiac diseases. Regarding the heart, the utility of SW elasticity was investigated in animal models^10–12^. Pernot et al. reported that SW elasticity could be used to measure myocardial stiffness in isolated rat hearts^10^. They also reported that SW elasticity differentiated between stiff, noncompliant infarcted myocardial walls and softer walls containing stunned myocardium in ischemic hearts of an ovine model, showing that SW elasticity was higher in infarcted myocardium^12^. In recent years, the feasibility of SW elasticity was investigated in human subjects^13,22,23^. Villemain et al. reported that SW elasticity could assess myocardial stiffness quantitatively, showing that SW elasticity was higher in patients with hypertrophic cardiomyopathy than in healthy volunteers^13^. Pislaru et al. reported that higher SW elasticity was observed in patients with cardiac amyloidosis^22^. Therefore, SW elasticity could be useful in evaluating cardiomyopathy. However, the utility of SW elasticity for assessing the severity of hypertensive LV hypertrophy remains unclear. The relationship of SW elasticity with myocardial histological tissue has not been well investigated.

To clarify these findings, we used hypertensive model rats and evaluated the efficacy of SW elasticity for histologically assessing myocardial hypertrophy. In the present study, SW elasticity was higher in hypertensive rats. Larger cross-sectional area of cardiomyocytes was observed in hypertensive rats, and SW elasticity was correlated with the cross-sectional area of cardiomyocytes. Our findings suggest that SW elasticity can evaluate the severity of hypertensive LV hypertrophy. SW elasticity may be able to detect early changes in hypertensive heart disease. Additionally, in the myocardium of hypertensive rats, myocardial fibrosis was slight, which was similar to that in control rats. SW elasticity has been reported to be correlated with fibrosis^4,5^, whereas the present study found that SW elasticity was related to myocardial hypertrophy in the myocardium with less fibrosis.

Cvijic et al. demonstrated that SW velocity—as measure of myocardial stiffness—was higher in patients with concentric hypertrophy than in those with concentric remodelling or healthy controls, indicating that the differences in SW velocity reflected intrinsic material properties^24^. Animal experiments suggest that LV hypertrophy alone cannot explain myocardial stiffening, but that the deposition of collagen fibres and qualitative changes of the collagen are major determinants^25,26^. SW elasticity may be influenced not only by myocardial hypertrophy but also by these myocardial properties.

### Clinical application

SW imaging is a non-invasive and quantitative method. The present study suggests that SW elasticity can provide valuable information for assessing the severity of LV hypertrophy. Additionally, SW elasticity may be useful as a clinical tool for assessing treatment effects for LV hypertrophy. SW elasticity may have the potential for identifying myocardial tissue characteristics, leading to the differentiation of cardiac diseases. Further studies are needed to determine the usefulness of SW elasticity. For clinical application of SW imaging, it is necessary to remove the influence of the beating of heart on SW velocity. A few recent studies have reported the feasibility of SW imaging under the beating hearts^13,27,28^. Villemain et al. have shown that SW imaging can quantify myocardial stiffness in human subjects^13^. Petrescu et al. also suggest that SW imaging has the potential to become a valuable method for the assessment of myocardial properties in human subjects^27,28^. The application seems to be possible.

### Limitations

There are limitations in the present study. First, this study evaluated SW elasticity in non-beating hearts, because the heart rate of rats is too rapid to obtain SW imaging. This study evaluated SW elasticity under a retrograde perfusion system to make the heart fully relaxed for simulating the end-diastolic phase. Second, SW elasticity was measured in the long-axis view, whereas myocardial histology was assessed in the short-axis view. Third, the myocardial stiffness and the structural properties are not homogenous in the left ventricle. It was not clear if SW elasticity was measured in the same area as histological evaluation. Fourth, histological evaluation, such as the cross-sectional area of cardiomyocytes and the extent of fibrosis, might not be exactly. However, the cross-sectional area of cardiomyocytes was measured at many locations, and interstitial fibrosis was automatically measured using computer-assisted image analysis. Fifth, the amount of interstitial fibrosis was small in hypertension rats. It was difficult to assess the correlation of SW elasticity with myocardial fibrosis. Finally, this study evaluated SW elasticity in the left ventricle. The effects of SW elasticity in the right ventricle were not assessed.

## Conclusions

SW elasticity was higher in hypertensive rats. SW elasticity was closely correlated with the degree of myocardial hypertrophy of left ventricle. Our findings suggest that SW elasticity has the potential for estimating the severity of hypertensive LV hypertrophy.

## Supplementary Information

Supplementary Legends.

Supplementary Figure S1.

Supplementary Figure S2.

Supplementary Figure S3.
