# Supplementary material for: Efficacy of shear wave elasticity for evaluating myocardial hypertrophy in hypertensive rats
Source: Sci Rep. 2021 Nov 24;11:22812. doi: 10.1038/s41598-021-02271-6 (PMC8613270; doi:10.1038/s41598-021-02271-6)
Supplement: Supplementary file 1 — Supplementary Legends. [file 41598_2021_2271_MOESM1_ESM.doc]

**Figure legends**

Supplementary Figure S1. Transthoracic echocardiography

(A) Transthoracic echocardiographic records in a hypertension rat. Left ventricular end-systolic and end-diastolic diameters were 8.3 mm and 6.0 mm. Interventricular septum and left ventricular free wall thickness were 1.4 mm and 1.5 mm. (B) Transthoracic echocardiographic records in a control rat. Left ventricular end-systolic and end-diastolic diameters were 6.9 mm and 3.3 mm. Interventricular septum and left ventricular free wall thickness were 1.1 mm and 1.2 mm.

Supplementary Figure S2. Histological evaluation of cardiomyocytes

Sections stained with hematoxylin-eosin of a hypertension rat (A) and a control rat (B) were shown. Histological findings were evaluated on left ventricular free wall indicated by white square. The cross-sectional area of cardiomyocytes was measured in the regions containing cellular nucleus using Image J software (yellow area). The cross-sectional area of cardiomyocytes was 421 μm^2^ in a hypertension rat (C) and 253 μm^2^ in a control rat (D). Scale bar indicated 100 μm.

Supplementary Figure S3. Histological evaluation of fibrosis

Sections stained with picrosirius red of a hypertension rat (A) and a control rat (B) were shown. Histological findings were evaluated on left ventricular free wall indicated by white square. Interstitial fibrosis was calculated using computer-assisted image analysis, and the percentage of fibrosis was 2.2% in a hypertension rat (C) and 1.5% in a control rat (D). Scale bar indicated 100 μm.
