# Supplementary figures and images for: Efficacy of shear wave elasticity for evaluating myocardial hypertrophy in hypertensive rats
Source: Sci Rep. 2021 Nov 24;11:22812. doi: 10.1038/s41598-021-02271-6 (PMC8613270; doi:10.1038/s41598-021-02271-6)

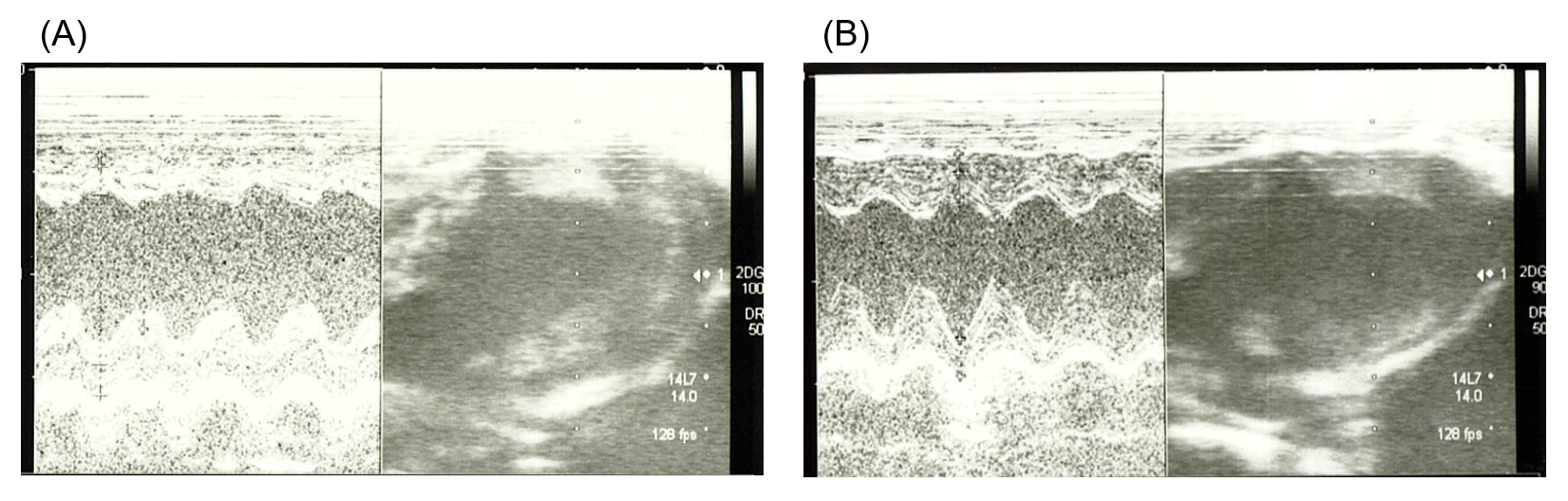

Supplement: Supplementary file 2 — Supplementary Figure S1. [file 41598_2021_2271_MOESM2_ESM.tif]

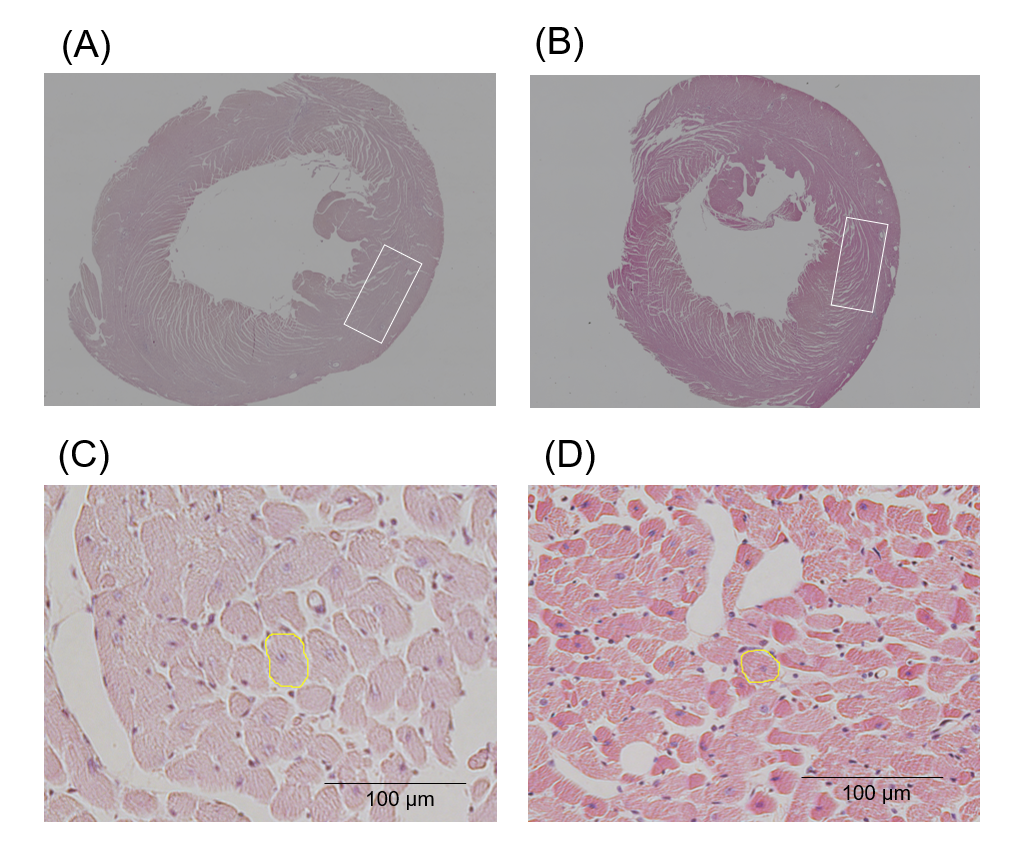

Supplement: Supplementary file 3 — Supplementary Figure S2. [file 41598_2021_2271_MOESM3_ESM.tif]

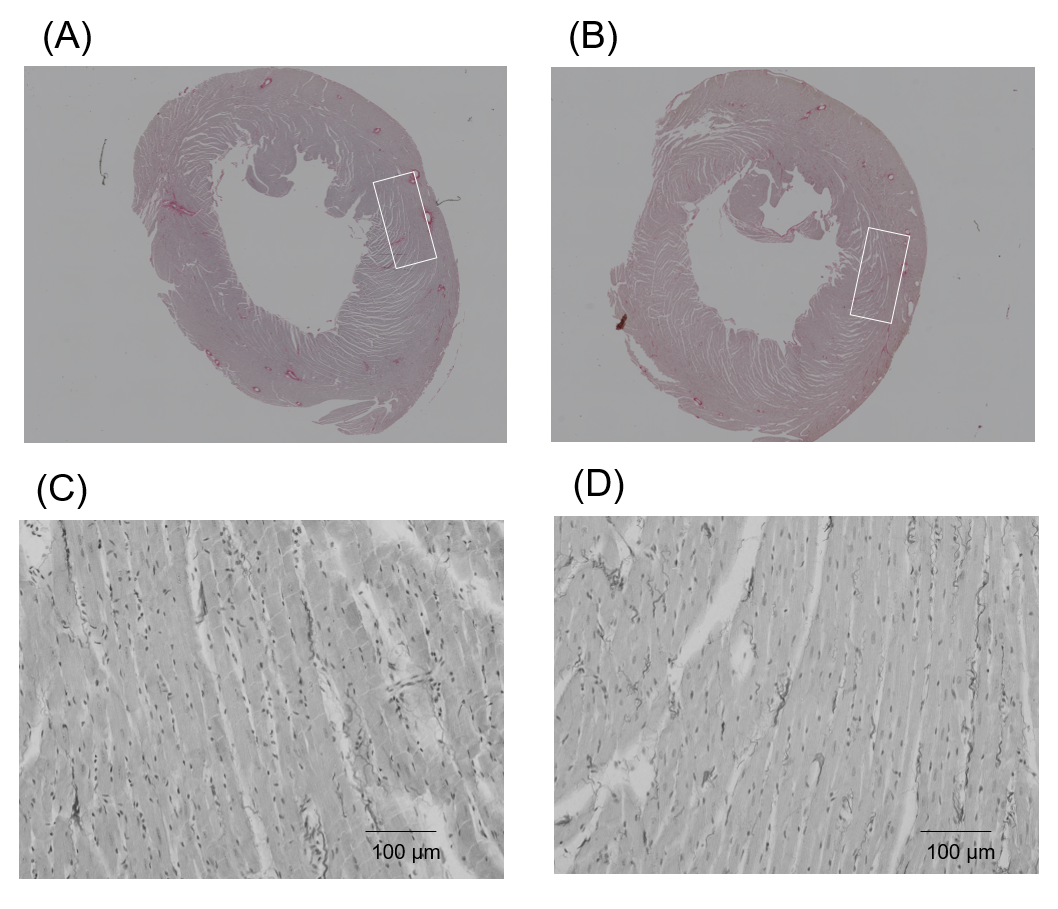

Supplement: Supplementary file 4 — Supplementary Figure S3. [file 41598_2021_2271_MOESM4_ESM.tif]
